# Transdiagnostic and disease-specific abnormalities in the default-mode network hubs in psychiatric disorders: A meta-analysis of resting-state functional imaging studies

**DOI:** 10.1192/j.eurpsy.2020.57

**Published:** 2020-05-29

**Authors:** Gaelle E. Doucet, Delfina Janiri, Rebecca Howard, Madeline O’Brien, Jessica R. Andrews-Hanna, Sophia Frangou

**Affiliations:** 1 Department of Psychiatry, Icahn School of Medicine at Mount Sinai, New York, New York, USA; 2 Brain Architecture, Imaging and Cognition Lab, Boys Town National Research Hospital, Omaha, Nebraska, USA; 3 Department of Neurology and Psychiatry, Sapienza University of Rome, Rome, Italy; 4 Graduate School of Biomedical Sciences, Icahn School of Medicine at Mount Sinai, New York, New York, USA; 5 Department of Psychology, University of Arizona, Tucson, Arizona, USA; 6 Evelyn F. McKnight Brain Institute, University of Arizona, Tucson, Arizona, USA; 7 Cognitive Science, University of Arizona, Tucson, Arizona, USA; 8 Centre for Brain Health, University of British Columbia, Vancouver, British Columbia, Canada

**Keywords:** Default mode network, meta-analysis, psychiatric disorders, resting-state fMRI studies

## Abstract

**Background.:**

The default mode network (DMN) dysfunction has emerged as a consistent biological correlate of multiple psychiatric disorders. Specifically, there is evidence of alterations in DMN cohesiveness in schizophrenia, mood and anxiety disorders. The aim of this study was to synthesize at a fine spatial resolution the intra-network functional connectivity of the DMN in adults diagnosed with schizophrenia, mood and anxiety disorders, capitalizing on powerful meta-analytic tools provided by activation likelihood estimation.

**Methods.:**

Results from 70 whole-brain resting-state functional magnetic resonance imaging articles published during the last 15 years were included comprising observations from 2,789 patients and 3,002 healthy controls.

**Results.:**

Specific regional changes in DMN cohesiveness located in the anteromedial and posteromedial cortex emerged as shared and trans-diagnostic brain phenotypes. Disease-specific dysconnectivity was also identified. Unmedicated patients showed more DMN functional alterations, highlighting the importance of interventions targeting the functional integration of the DMN.

**Conclusion.:**

This study highlights functional alteration in the major hubs of the DMN, suggesting common abnormalities in self-referential mental activity across psychiatric disorders.

## Introduction

The default mode network (DMN) refers to a set of brain regions that are more active in conditions unconstrained by explicit task instructions [1, 2], commonly termed “resting-state” conditions. The DMN gained prominence in cognitive neuroscience through the seminal work of Raichle and colleagues, who coined the term “default mode” [3], and that of Greicius et al. [4], who established the presence of this network during resting-state functional magnetic resonance imaging (rsfMRI). The DMN has been shown to be reproducible [5–8] and has relatively low inter- and intra-subject variability [7–9] across different acquisition and analysis protocols [5, 10]. Brain regions reliably identified as part of the DMN include the medial prefrontal cortex (MPFC)/anterior cingulate cortex (ACC), the precuneus/posterior cingulate cortex (PCu/PCC), the angular gyrus, and regions of the medial and lateral temporal cortex [3,11–14]. Among these regions, the MPFC and the posteromedial cortex (PMC), which includes the PCu and PCC, are often considered core components of the network, showing widespread patterns of connectivity and activity across a range of internally directed tasks/processes [13,15,16].

The DMN has been associated with wide array of cognitive functions including self-referential mental activity [11,17,18] (particularly self-monitoring and autobiographical thoughts [19], stimulus-independent thought [1,13,20], and predictive planning [17,19]). Available evidence suggests that different DMN regions may contribute preferentially to the varied functions attributed to the DMN. Notably, the MPFC has been linked to the processing of visceromotor reactions elicited by self-referential information [21,22], while the PMC has been implicated in the monitoring of the external and internal environment and in self-related mental representations during spontaneous cognition [13,23,24].

Although DMN dysfunction has emerged as a consistent biological correlate of multiple neuropsychiatric disorders [11,25–27], the focus of this paper is on the role of the DMN in psychiatric disorders. Specifically, better understanding of the intra-network functional connectivity (i.e., cohesiveness) of the DMN may shed light on the pathophysiology of psychiatric disorders and reveal targets for improving brain network synchrony with therapeutic potential. A number of key studies have recently synthesized the available evidence on DMN cohesiveness in the major psychiatric disorders. A review of the relevant literature in schizophrenia not only highlighted significant interstudy variation, but also identified increased functional connectivity between the ventral MPFC and other DMN regions as the most common finding [28]; these results were considered relevant to the self-referential nature of psychotic symptoms and the poor insight that are common features of this disorder [28]. In major depressive disorder (MDD), a meta-analysis of 25 studies provided evidence of increased functional connectivity between the MPFC and medial temporal DMN regions centered on the hippocampus, which is thought to underpin affective self-referential cognitions that are typical of MDD [29]. This regional pattern of hyperconnectivity may be nested within a more widespread reduction in DMN cohesiveness as indicated by a large-scale study involving rsfMRI data from 1,300 depressed patients and 1,128 healthy individuals [30]. In obsessive–compulsive disorder (OCD), meta-analysis of 18 studies identified the ventral MPFC and the ACC as the two DMN regions with abnormal functional connectivity with the rest of the network regions [31]; this pattern of dysconnectivity has been linked to patients’ difficulties in switching away from self-generated obsessive thoughts and compulsive actions. In post-traumatic stress disorder (PTSD), a meta-analysis of 14 studies showed decreased connectivity within the DMN of the PCC, posterior hippocampus, and ventral MPFC [32], which could be associated with patients’ preoccupation with autobiographical information. On the other hand, a meta-analysis of 20 studies on patients with anxiety disorders [33] and a review of 23 studies in bipolar disorder (BD) [34] did not produce evidence for alternations in the intra-network functional connectivity of the DMN. Finally, a meta-analysis of 242 rsfMRI studies covering schizophrenia, MDD, BD, OCD, PTSD, anxiety disorders, and childhood neurodevelopmental disorders identified hypoconnectivity of the MPFC as a common transdiagnostic feature of intra-network DMN dysconnectivity [26].

The aim of this study was to examine the intra-network connectivity of the DMN specifically in adults diagnosed with schizophrenia, mood or anxiety disorders. The focus on schizophrenia, mood and anxiety disorders was predicated on their overlapping clinical features, frequent serial or concurrent comorbidity [35], and highly correlated profiles of task-related abnormalities in brain functional activation [36,37]. Accordingly, we capitalized on powerful meta-analytic tools provided by activation likelihood estimation (ALE) [38–41] to analyze data from whole-brain resting-state connectivity studies published during the last 15 years that examined the functional cohesiveness of the DMN in these disorders. Based on the available evidence, our working hypotheses were that (a) alterations in DMN cohesiveness in adults across the major psychiatric disorders would implicate the major hubs of the network centered in the anteriomedial cortex and PMC and (b) our analyses would uncover novel information about disease-specific dysconnectivity in the regional pattern of the DMN.

## Method

### Literature search and article eligibility

We applied the Preferred Reporting Items for Systematic Reviews and Meta-analyses criteria (http://www.prisma-statement.org/) (Figure S1) to identify rsfMRI whole-brain studies published between January 1, 2005 and January 31, 2019 that reported significant differences in DMN intra-network connectivity between healthy adults and adult patients diagnosed with schizophrenia, MDD, BD, anxiety disorders, OCD, or PTSD (in the range 18–65 years old). In addition, we required that (a) in each study the spatial composition of the DMN was defined according to Raichle et al. [3] and Buckner et al. [11]; (b) the DMN was extracted by independent component analysis (ICA) or by using a DMN region as a seed; (c) the temporal correlations between the time-series of distinct DMN brain regions were computed in a time-locked fashion over the entire resting-state scan thus yielding static, nonshifted functional connectivity measures; and (d) studies used a whole-brain analysis approach. Studies using other functional measures such as dynamic functional connectivity, amplitude of low-frequency fluctuation, regional homogeneity, graph-theory, or effective connectivity were not included (further details on search strategy and article eligibility in eMethods).

### Coding and database construction

From each selected study, we extracted coordinates of significant case–control differences in the functional connectivity among DMN regions as we were interested in DMN functional cohesiveness. When necessary, the coordinates of the primary studies were converted from the Montreal Neurological Institute space to Talairach space using the icbm_other2tal transformation. These coordinates were coded by clinical diagnosis and by the direction of change; hyperconnectivity was defined as increased positive functional connectivity in the patient group relative to the healthy control group, and hypoconnectivity was defined as decreased positive functional connectivity in the patient group relative to the healthy control group. Additionally, coordinates of case–control differences were coded according to the strength of the magnetic field of the scanner, the analytical method used to compute interregional correlations (ICA or seed-based analyses), and according to whether participants were instructed to keep their eyes open or closed during data acquisition. Separately for patient and control groups, we coded sample size, age, and sex (percentage male). In patients only, (a) medication status in each study was coded as the percentage of patients’ sample receiving any psychotropic medication and (b) ratings of psychopathology from the different clinical instruments used in the primary studies were coded as “minimal,” “mild,” “moderate,” and “severe” to enable analyses across clinical populations. Further details of the database construction are provided in the Supplementary eMethods.

### Activation likelihood estimation

We used ALE [38–41] implemented in MATLAB, to test whether the whole-brain coordinates of case–control differences across studies converged into discrete clusters with a nonrandom spatial distribution. Per best practice standards [38–41], clusters of aberrant connectivity were identified using a cluster-level familywise-error-corrected threshold of *p* < 0.05 (cluster-forming threshold at voxel-level *p* < 0.001). Subsequently, for each cluster of significant DMN dysconnectivity, we extracted the per-voxel probability of case–control differences from the modeled functional connectivity maps. We then calculated the contribution of age, sex, symptomatic severity, acquisition (eyes closed/open), analyses parameters (ICA/seed-based), and location of the seed (Pcu/PCC, ACC/MPFC, lateral parietal gyrus, lateral temporal gyrus, and medial temporal cortex) using nonparametric Kruskal–Wallis tests and Spearman correlations as appropriate.

To identify clusters of transdiagnostic changes, we performed an ALE analysis on the pooled coordinates of aberrant DMN intra-network connectivity, regardless of the direction of change, across all diagnoses. This global analysis on transdiagnostic aberrant brain connectivity has the advantage that it provides an optimal summary of neuroimaging findings across all disorders [42]. Additional diagnosis-specific analyses were conducted (a) using the coordinates of case–control differences in DMN connectivity from studies that included only unmedicated patients and (b) using the coordinates of case–control differences in DMN hypo- and hyperconnectivity in each diagnostic category separately.

### Ancillary analyses

In order to ensure that the disorder contribution to the transdiagnostic clusters was not driven by the overrepresentation of MDD and schizophrenia in literature (see Table 1 and Table S1), we further applied a normalization procedure to the % contribution extracted for the disorders. Specifically, a normalization score was calculated for each disorder by dividing the % contribution of the disorder to the transdiagnostic cluster with the percentage of included studies related to this disorder. Values higher than 1 indicate an overrepresentation of this disorder.

**Table 1.** Samples included in the database.

Diagnostic coding	Studies	Patients				Healthy individuals		
		Sample (*N*)	Age	Male sex (%)	Medicated (%)	Sample (N)	Age	Male sex (%)
Schizophrenia	31	1,486	32.54 (7.03)	65 (14)	72 (40)	1,814	31.94 (6.47)	58 (14)
Major depressive disorder	22	779	33.30 (8.68)	36 (10)	23 (38)	688	32.73 (8.76)	41 (10)
Bipolar disorder	4	191	33.04 (7.41)	47 (9)	49 (49)	202	34.93 (6.35)	47 (9)
Obsessive compulsive disorder	5	121	27.40 (2.64)	55 (10)	16 (24)	116	26.67 (3.19)	49 (12)
Post-traumatic stress disorder	7	192	35.04 (7.29)	65 (32)	11 (17)	163	33.59 (8.11)	69 (27)
Social anxiety disorder	1	20	22.90 (3.99)	70 (0)	0 (0)	19	21.89 (3.77)	74 (0)

Values age, percentage (%) of male, and percentage (%) of medicated are shown as mean (standard deviation); age in years.

## Results

### Studies and coordinates

In total, 70 articles were selected, comprising observations from 2,789 patients and 3,002 healthy individuals (Table 1). Full citations and details of the selected articles are provided in Table S1. There were no case–control differences in age (*p* = 0.05) or sex (*p* = 0.65). However, there was a significant effect of diagnosis (*χ* ^2^ = 17.8; *p* = 1.4 × 10^−4^). Diagnosis-specific ALE analyses were only performed for schizophrenia (number of studies: 31) and MDD (number of studies: 22); the data for all other disorders were insufficient as the minimum number of studies for robust results is currently considered to be 17 [40].

### Activation likelihood estimation

#### DMN regions showing aberrant connectivity across all diagnoses

**Figure 1.**
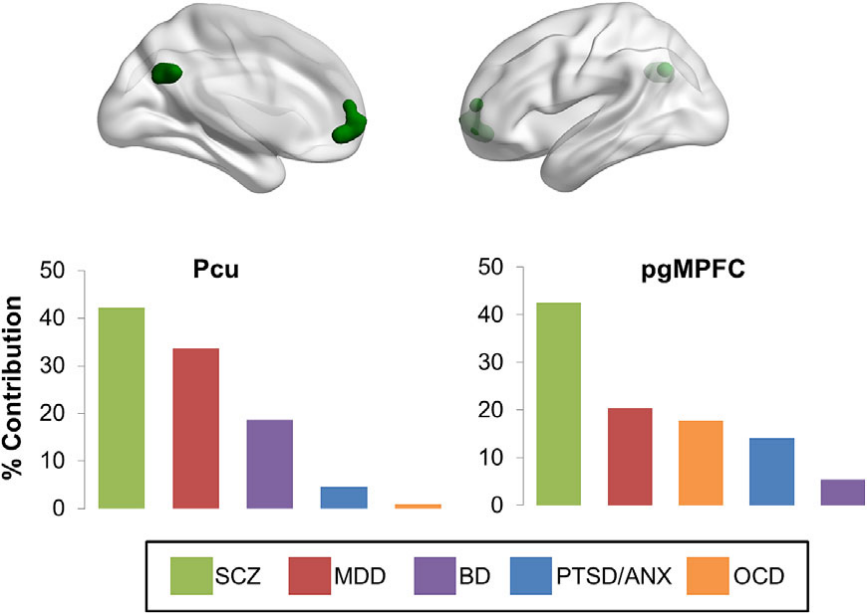
Transdiagnostic clusters of aberrant connectivity in the default mode network. Abbreviations: ANX, social anxiety disorder; BD, bipolar disorder; MDD, major depressive disorder; OCD, obsessive compulsive disorder; Pcu, precuneus; pgMPFC, perigenual medial prefrontal cortex; PTSD, post-traumatic stress disorder; SCZ, schizophrenia.

Coordinates of aberrant connectivity within the DMN clustered in the left perigenual medial prefrontal cortex (pgMPFC) (peak coordinates: *x* = −2, *y* = 56, and *z* = −2; 295 voxels) and in the PCu (peak coordinates: *x* = 0, *y* = −58, *z* = 32; 254 voxels) (Figure 1). Coordinates from studies on schizophrenia contributed the most in both regions (pgMPFC: 42%, PCu: 42%), followed by MDD (pgMPFC: 20%, PCu: 34%). The other disorders showed limited contribution to either cluster (<20%). The contribution of coordinates reflecting hypo- or hyper-connectivity was relatively similar for each cluster (pgMPFC: 53 vs. 47%, PCu: 67 vs. 33%). No significant effects were found for the moderator variables examined: age, sex, symptom severity, acquisition, and analyses parameters (all uncorrected *p* > 0.05).

Importantly, the relative high contribution of schizophrenia and MDD to these two clusters was not related to the overrepresentation of the studies on MDD and schizophrenia included in the analyses. The normalization scores of the % contribution for both disorders and clusters ranged between 0.65 and 1.05.

When we restricted the ALE analysis to coordinates from studies from patients who were unmedicated at the time of the scanning (schizophrenia: *n* = 7; MDD: *n* = 15; BD: *n* = 3; OCD: *n* = 3; PTSD: *n* = 4; social anxiety disorder: *n* = 1), we identified three clusters located in the pgMPFC (*x* = 0, *y* = 60, *z* = −4, *k* = 182 voxels), the dmPFC (*x* = −12, *y* = 54, 28, *k* = 122 voxels), and the angular gyrus (*x* = 42, *y* = −64, *z* = 44, *k* = 93 voxels) (Figure S2). The pgMPFC cluster included mostly contribution from PTSD (47%), followed by schizophrenia (31%) that mostly reflected hypoconnectivity (63%). The dmPFC cluster included mainly contributions from MDD (47%) followed by schizophrenia (30%) reflecting hyperconnectivity (99.6%). The cluster in the right angular gyrus included mainly contributions from studies on MDD (77%) with equal contribution of hypo- and hyperconnectivity (55 vs. 45%). No significant moderator effects were found for age, sex, symptom severity, acquisition, and analyses parameters (all uncorrected *p* > 0.05).

#### DMN regions showing aberrant connectivity in schizophrenia

**Figure 2.**
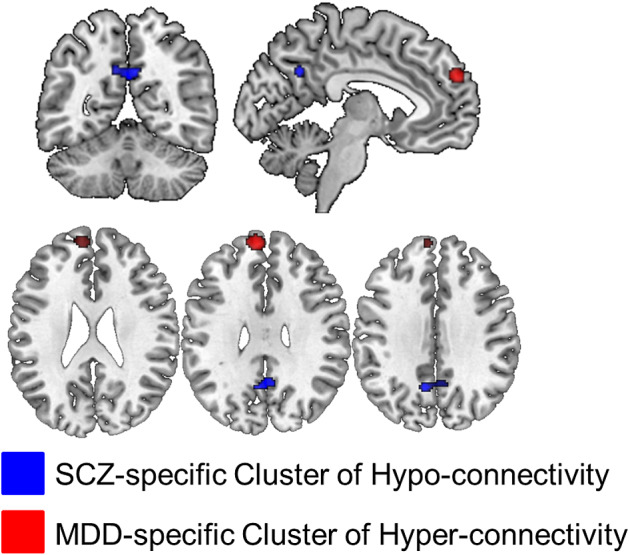
Disease-specific clusters of aberrant connectivity in the default mode network. Abbreviations: MDD, major depressive disorder; SCZ, schizophrenia.

Results emerging from the schizophrenia group alone reinforced the role of PCu in schizophrenia where hypoconnectivity was further confirmed in a cluster which overlapped with the corresponding cluster in the transdiagnostic analysis *(*peak coordinates: *x* = −2, *y* = −56, *z* = 32; 79 voxels) (Figure 2). No significant moderator effects were found for age, sex, symptom severity, acquisition, and analyses parameters (all uncorrected *p* > 0.05).

#### DMN regions showing aberrant connectivity in MDD

Within the MDD group, an additional cluster of hyperconnectivity emerged in the left dmPFC (peak coordinates: *x* = −8, *y* = 56, *z* = 30; 99 voxels) (Figure 2). No significant moderator effects were found for age, sex, symptom severity, acquisition, and analyses parameters (all uncorrected *p* > 0.05).

## Discussion

We conducted a large-scale meta-analysis on the intra-network DMN functional connectivity comprising data from 70 rsfMRI studies derived from 2,789 patients with schizophrenia, mood and anxiety disorders and 3,002 healthy controls. We specifically focused on the DMN because this network has been shown to be the most reliable network among the major resting-state networks [5–8]. Case–control difference in this network functional connectivity may be therefore less sensitive to the high variability in acquisition and analysis protocols across studies [43]. In accordance with our hypothesis, the results implicated the major hubs of the DMN located in the anteromedial cortex and PMC and suggest that abnormalities in self-referential mental activity are common across these major psychiatric disorders. The posteromedial cluster corresponded to the PCu and showed evidence of dysconnectivity within the DMN across all diagnoses, as well as specific hypoconnectivity for schizophrenia. The pattern observed in the anteromedial cortex was more complex; the pgMPFC appeared to show abnormal intra-network connectivity across diagnoses while the dmPFC showed hyperconnectivity which was specific to MDD. The results of this study highlight the importance of the anteromedial cortex and of the PMC, for psychiatric disorders, and reinforce their known function as hub regions of the DMN [11,13,15,44,45] and the brain more generally [46–48].

It is well known that MPFC is functionally diverse [48–51]. In describing the MPFC subregions implicated here, we follow the functional parcellation proposed by de la Vega and colleagues [49], which largely overlaps with the multimodal parcellation put forward by Glasser et al. [51]. De la Vega et al. [52] mapped mental operations to MPFC subregions following functional parcellation based on patterns of connectivity derived from a meta-analysis of approximately 10,000 studies available in the Neurosynth neuroimaging database. Their findings confirmed prior reports linking the anterior MPFC to affective and social processes and value-based decision-making, but the degree of engagement by these processes differed in MPFC subregions. Social cognition (comprising social perception, self-referential thought, and mentalizing) was maximally associated with the dmPFC, followed by the pgMPFC and then the ventromedial prefrontal cortex.

The PMC has been associated with multiple mental processes [13,23,24]. In addressing the question of the relative functional specificity of the PCu, we draw on the work of Bzdok et al. [53] who capitalized on the large functional imaging database provided by the BrainMap platform to define functional profile of the PCu. They found that the PCu, as well as most PMC regions, was significantly associated with mental operations relating to social cognition, theory of mind, and episodic memory (including autobiographic memory). Notably, mental operations relating to cognitive control, including attentional engagement, inhibition, reasoning, and orienting responses, were distinctly associated with the PCu [53]. This finding confirms prior observations that attenuation of PCu activity during the transition from rest to task enables the engagement of sustained mechanism related to attention and working memory that are required for successful task performance [1].

The dysconnectivity profiles identified are likely to influence the functional integration of the PCu and MPFC subregions with networks outside the DMN as predicted by their high level of functional and anatomical connectivity across the brain [15,44,54]. In the context, DMN cohesiveness, the preferential functional profiles of the regions identified in the current meta-analysis points to disrupted integration between affective processing/value-based decision making, social cognition, and cognitive control. The differential regional dysconnectivity observed goes a long way toward reconciling conflicting results from prior studies particularly those on schizophrenia [28] and MDD [29,30], which also contributed the majority of the data analyzed here. Of note, the analyses conducted separately in each disorder also highlighted the importance of the dmPFC hyperconnectivity for MDD. This region is considered a nexus of clinical significance for patients with MDD [55], especially those with elevated levels of rumination [56]. Relatedly, increased dMPFC connectivity with other prefrontal regions has been shown to be associated with higher levels of negative self-referential cognition [57] while increased dMPFC connectivity with the PCC post-electroconvulsive therapy may be conducive to improved clinical response [58].

Abnormalities in affect [59], valuation [60], self-referential [27,61], and social cognition [62–64] and cognitive control [65,66] represent domain-general dimensions that have been consistently implicated in all the disorders considered here. We acknowledge that psychiatric disorders also show marked variations in their clinical presentation both between diagnoses and between individuals with the same diagnosis. The current results suggest that mapping DMN regional dysconnectivity is also domain-general and likely to underpin variable within-domain deficits in different individuals and diagnoses. This issue should be explored further in large single samples studies where both clinical, cognitive and connectivity measures can be examined simultaneously.

This study has several strengths and limitations. The number of articles selected in this meta-analysis was lower than that in prior meta-analyses on individual disorders [29–34] because we considered only rsfMRI studies in adults and among those only studies focusing on the intra-network cohesiveness of the DMN. The restriction on adult individuals was predicated by age-related impact on functional connectivity that could have biased results [67,68]. We included studies that used both ICA and seed-based analyses to compute connectivity and found that the results were robust to this methodological variation. The concern with using seed-based analyses only is that the interstudy variability in the exact localization of the seed may influence the pattern of connectivity; however, there is evidence that this is not the case [32]. The number of studies in BD, PTSD, OCD, and anxiety disorders describing significant case–control differences in the DMN was very small, and hence we were unable to undertake diagnosis-specific analyses for these disorders. This is a general limitation of the ALE method, which does not account for negative results. Furthermore, the use of different acquisition protocols and statistical thresholds in the included studies could influence measurement reliability [43]. In this regard, any meta-analysis is prone to publication bias, as we cannot control for statistical methodologies used in the original studies for thresholding the results. However, this is an issue that is out of the scope of this study. Further effort to optimize analytic strategies for reliability should be done across clinical studies [43]. Nevertheless, we found that the transdiagnostic results were not influenced by the larger number of studies in schizophrenia and MDD and were in line with previous transdiagnostic studies [26,69]. The cross-sectional nature of the studies included does not allow us to comment on the evolution of these patterns of connectivity and their association with disease stage. We addressed the issue of medication by undertaking a sub-analysis on coordinates derived from studies with unmedicated patients. The majority of these studies involved patients with MDD, and the results obtained appear more pertinent to this disorder. Our findings suggest that medication may have an effect on DMN connectivity that may be most consistently observed in the angular gyrus which is in line with previous reports on the “normalizing” effect of antidepressant medication on hyperconnectivity of this region [70]. It is also in line with the study by Schilbach et al. [69] that described that medicated patients with MDD and SCZ showed hypoconnectivity in the lateral parietal cortex of the DMN.

Overall, this meta-analysis identified regional changes in DMN cohesiveness in the anterior and posterior hubs of the DMN as shared and specific brain phenotypes of psychiatric disorders. These brain phenotypes have the potential to serve as targets for interventions aiming to improve the functional integration of the DMN across diverse psychiatric populations. The clusters found in this meta-analysis are freely available (see Supplementary Material) and can then be used as a priori regions of interest for future studies.

## Data Availability

Default mode network clusters of dysconnectivity resulting from the meta-analyses are freely available for download (see Supplementary Material).

## Supplementary material

For supplementary material accompanying this paper visit http://dx.doi.org/10.1192/j.eurpsy.2020.57. click here to view supplementary material

## References

[1] Mazoyer B, Zago L, Mellet E, Bricogne S, Etard O, Houde O, et al. Cortical networks for working memory and executive functions sustain the conscious resting state in man. Brain Res Bull. 2001;54:287–298. 11287133 10.1016/s0361-9230(00)00437-8

[2] Shulman GL, Fiez JA, Corbetta M, Buckner RL, Miezin FM, Raichle ME, et al. Common blood flow changes across visual tasks: II. Decreases in cerebral cortex. J Cogn Neurosci. 1997;9:648–663. 23965122 10.1162/jocn.1997.9.5.648

[3] Raichle ME, MacLeod AM, Snyder AZ, Powers WJ, Gusnard DA, Shulman GL. A default mode of brain function. Proc Natl Acad Sci U S A. 2001;98:676–682. 11209064 10.1073/pnas.98.2.676 PMC14647

[4] Greicius MD, Krasnow B, Reiss AL, Menon V. Functional connectivity in the resting brain: a network analysis of the default mode hypothesis. Proc Natl Acad Sci U S A. 2003;100:253–258. 12506194 10.1073/pnas.0135058100 PMC140943

[5] Doucet GE, Lee WH, Frangou S. Evaluation of the spatial variability in the major resting-state networks across human brain functional atlases. Hum Brain Mapp. 2019;40:4577–4587. 31322303 10.1002/hbm.24722 PMC6771873

[6] Gordon EM, Laumann TO, Adeyemo B, Huckins JF, Kelley WM, Petersen SE. Generation and evaluation of a cortical area parcellation from resting-state correlations. Cereb Cortex. 2016;26:288–303. 25316338 10.1093/cercor/bhu239 PMC4677978

[7] Yeo BT, Krienen FM, Sepulcre J, Sabuncu MR, Lashkari D, Hollinshead M, et al. The organization of the human cerebral cortex estimated by intrinsic functional connectivity. J Neurophysiol. 2011;106:1125–1165. 21653723 10.1152/jn.00338.2011 PMC3174820

[8] Damoiseaux JS, Rombouts SA, Barkhof F, Scheltens P, Stam CJ, Smith SM, et al. Consistent resting-state networks across healthy subjects. Proc Natl Acad Sci U S A. 2006;103:13848–13853. 16945915 10.1073/pnas.0601417103 PMC1564249

[9] Meindl T, Teipel S, Elmouden R, Mueller S, Koch W, Dietrich O, et al. Test-retest reproducibility of the default-mode network in healthy individuals. Hum Brain Mapp. 2010;31:237–246. 19621371 10.1002/hbm.20860 PMC6871144

[10] Marchitelli R, Collignon O, Jovicich J. Test–retest reproducibility of the intrinsic default mode network: influence of functional magnetic resonance imaging slice-order acquisition and head-motion correction methods. Brain Connect. 2017;7:69–83. 28084793 10.1089/brain.2016.0450

[11] Buckner RL, Andrews-Hanna JR, Schacter DL. The brain's default network: anatomy, function, and relevance to disease. Ann N Y Acad Sci. 2008;1124:1–38. 18400922 10.1196/annals.1440.011

[12] Raichle ME. The brain's default mode network. Annu Rev Neurosci. 2015;38:433–447. 25938726 10.1146/annurev-neuro-071013-014030

[13] Andrews-Hanna JR, Reidler JS, Sepulcre J, Poulin R, Buckner RL. Functional-anatomic fractionation of the brain's default network. Neuron. 2010;65:550–562. 20188659 10.1016/j.neuron.2010.02.005 PMC2848443

[14] Margulies DS, Ghosh SS, Goulas A, Falkiewicz M, Huntenburg JM, Langs G, et al. Situating the default-mode network along a principal gradient of macroscale cortical organization. Proc Natl Acad Sci U S A. 2016;113:12574–12579. 27791099 10.1073/pnas.1608282113 PMC5098630

[15] Fransson P, Marrelec G. The precuneus/posterior cingulate cortex plays a pivotal role in the default mode network: evidence from a partial correlation network analysis. Neuroimage. 2008;42:1178–1184. 18598773 10.1016/j.neuroimage.2008.05.059

[16] Buckner RL, DiNicola LM. The brain's default network: updated anatomy, physiology and evolving insights. Nat Rev Neurosci. 2019;20:593–608. 31492945 10.1038/s41583-019-0212-7

[17] Spreng RN, Mar RA, Kim AS. The common neural basis of autobiographical memory, prospection, navigation, theory of mind, and the default mode: a quantitative meta-analysis. J Cogn Neurosci. 2009;21:489–510. 18510452 10.1162/jocn.2008.21029

[18] Gusnard DA, Akbudak E, Shulman GL, Raichle ME. Medial prefrontal cortex and self-referential mental activity: relation to a default mode of brain function. Proc Natl Acad Sci U S A. 2001;98:4259–4264. 11259662 10.1073/pnas.071043098 PMC31213

[19] Schacter DL, Addis DR, Buckner RL. Remembering the past to imagine the future: the prospective brain. Nat Rev Neurosci. 2007;8:657–661. 17700624 10.1038/nrn2213

[20] Fox KC, Spreng RN, Ellamil M, Andrews-Hanna JR, Christoff K. The wandering brain: meta-analysis of functional neuroimaging studies of mind-wandering and related spontaneous thought processes. Neuroimage. 2015;111:611–621. 25725466 10.1016/j.neuroimage.2015.02.039

[21] McGuire PK, Paulesu E, Frackowiak RS, Frith CD. Brain activity during stimulus independent thought. Neuroreport. 1996;7:2095–2099. 8930966

[22] Gusnard DA. Being a self: considerations from functional imaging. Conscious Cogn. 2005;14:679–697. 16256372 10.1016/j.concog.2005.04.004

[23] Cavanna AE, Trimble MR. The precuneus: a review of its functional anatomy and behavioural correlates. Brain. 2006;129:564–583. 16399806 10.1093/brain/awl004

[24] Margulies DS, Vincent JL, Kelly C, Lohmann G, Uddin LQ, Biswal BB, et al. Precuneus shares intrinsic functional architecture in humans and monkeys. Proc Natl Acad Sci U S A. 2009;106:20069–20074. 19903877 10.1073/pnas.0905314106 PMC2775700

[25] Greicius M. Resting-state functional connectivity in neuropsychiatric disorders. Curr Opin Neurol. 2008;21:424–430. 18607202 10.1097/WCO.0b013e328306f2c5

[26] Sha Z, Wager TD, Mechelli A, He Y. Common dysfunction of large-scale neurocognitive networks across psychiatric disorders. Biol Psychiatry. 2019;85:379–388. 30612699 10.1016/j.biopsych.2018.11.011

[27] Andrews-Hanna JR, Christoff K, O'Connor MF. Dynamic regulation of internal experience In: Lane R, Ryan L, Nadel L, editors. The neuroscience of enduring change: the neural basis of talk therapies. New York, NY: Oxford University Press; 2019.

[28] Hu ML, Zong XF, Mann JJ, Zheng JJ, Liao YH, Li ZC, et al. A review of the functional and anatomical default mode network in schizophrenia. Neurosci Bull. 2017;33:73–84. 27995564 10.1007/s12264-016-0090-1 PMC5567552

[29] Kaiser RH, Andrews-Hanna JR, Wager TD, Pizzagalli DA. Large-scale network dysfunction in major depressive disorder: a meta-analysis of resting-state functional connectivity. JAMA Psychiatry. 2015;72:603–611. 25785575 10.1001/jamapsychiatry.2015.0071 PMC4456260

[30] Yan CG, Chen X, Li L, Castellanos FX, Bai TJ, Bo QJ, et al. Reduced default mode network functional connectivity in patients with recurrent major depressive disorder. Proc Natl Acad Sci U S A. 2019;116:9078–9083. 30979801 10.1073/pnas.1900390116 PMC6500168

[31] Gursel DA, Avram M, Sorg C, Brandl F, Koch K. Frontoparietal areas link impairments of large-scale intrinsic brain networks with aberrant fronto-striatal interactions in OCD: a meta-analysis of resting-state functional connectivity. Neurosci Biobehav Rev. 2018;87:151–160. 29410103 10.1016/j.neubiorev.2018.01.016

[32] Koch SB, van Zuiden M, Nawijn L, Frijling JL, Veltman DJ, Olff M. Aberrant resting-state brain activity in posttraumatic stress disorder: a meta-analysis and systematic review. Depress Anxiety. 2016;33:592–605. 26918313 10.1002/da.22478

[33] Xu J, Van Dam NT, Feng C, Luo Y, Ai H, Gu R, et al. Anxious brain networks: a coordinate-based activation likelihood estimation meta-analysis of resting-state functional connectivity studies in anxiety. Neurosci Biobehav Rev. 2019;96:21–30. 30452934 10.1016/j.neubiorev.2018.11.005

[34] Syan SK, Smith M, Frey BN, Remtulla R, Kapczinski F, Hall GBC, et al. Resting-state functional connectivity in individuals with bipolar disorder during clinical remission: a systematic review. J Psychiatry Neurosci. 2018;43:298–316. 30125243 10.1503/jpn.170175 PMC6158027

[35] Plana-Ripoll O, Pedersen CB, Holtz Y, Benros ME, Dalsgaard S, de Jonge P, et al. Exploring comorbidity within mental disorders among a Danish national population. JAMA Psychiatry. 2019;76:259–70. 30649197 10.1001/jamapsychiatry.2018.3658 PMC6439836

[36] Janiri D, Moser DA, Doucet GE, Luber MJ, Rasgon A, Lee WH, et al. Shared neural phenotypes for mood and anxiety disorders: a meta-analysis of 226 task-related functional imaging studies. JAMA Psychiatry. 2019:1–8. 10.1001/jamapsychiatry.2019.3351 PMC6822098 31664439

[37] Sprooten E, Rasgon A, Goodman M, Carlin A, Leibu E, Lee WH, et al. Addressing reverse inference in psychiatric neuroimaging: meta-analyses of task-related brain activation in common mental disorders. Hum Brain Mapp. 2017;38:1846–1864. 28067006 10.1002/hbm.23486 PMC5347927

[38] Eickhoff SB, Bzdok D, Laird AR, Kurth F, Fox PT. Activation likelihood estimation meta-analysis revisited. Neuroimage. 2012;59:2349–2361. 21963913 10.1016/j.neuroimage.2011.09.017 PMC3254820

[39] Eickhoff SB, Laird AR, Grefkes C, Wang LE, Zilles K, Fox PT. Coordinate-based activation likelihood estimation meta-analysis of neuroimaging data: a random-effects approach based on empirical estimates of spatial uncertainty. Hum Brain Mapp. 2009;30:2907–2926. 19172646 10.1002/hbm.20718 PMC2872071

[40] Eickhoff SB, Nichols TE, Laird AR, Hoffstaedter F, Amunts K, Fox PT, et al. Behavior, sensitivity, and power of activation likelihood estimation characterized by massive empirical simulation. Neuroimage. 2016;137:70–85. 27179606 10.1016/j.neuroimage.2016.04.072 PMC4981641

[41] Turkeltaub PE, Eickhoff SB, Laird AR, Fox M, Wiener M, Fox P. Minimizing within-experiment and within-group effects in activation likelihood estimation meta-analyses. Hum Brain Mapp. 2012;33:1–13. 21305667 10.1002/hbm.21186 PMC4791073

[42] Muller VI, Cieslik EC, Serbanescu I, Laird AR, Fox PT, Eickhoff SB. Altered brain activity in unipolar depression revisited: meta-analyses of neuroimaging studies. JAMA Psychiatry. 2017;74:47–55. 27829086 10.1001/jamapsychiatry.2016.2783 PMC5293141

[43] Zuo XN, Xu T, Milham MP. Harnessing reliability for neuroscience research. Nat Hum Behav. 2019;3:768–771. 31253883 10.1038/s41562-019-0655-x

[44] Fox MD, Snyder AZ, Vincent JL, Corbetta M, Van Essen DC, Raichle ME. The human brain is intrinsically organized into dynamic, anticorrelated functional networks. Proc Natl Acad Sci U S A. 2005;102:9673–9678. 15976020 10.1073/pnas.0504136102 PMC1157105

[45] Fransson P. How default is the default mode of brain function? Further evidence from intrinsic BOLD signal fluctuations. Neuropsychologia. 2006;44:2836–2845. 16879844 10.1016/j.neuropsychologia.2006.06.017

[46] Buckner RL, Sepulcre J, Talukdar T, Krienen FM, Liu H, Hedden T, et al. Cortical hubs revealed by intrinsic functional connectivity: mapping, assessment of stability, and relation to Alzheimer's disease. J Neurosci. 2009;29:1860–1873. 19211893 10.1523/JNEUROSCI.5062-08.2009 PMC2750039

[47] Hagmann P, Cammoun L, Gigandet X, Meuli R, Honey CJ, Wedeen VJ, et al. Mapping the structural core of human cerebral cortex. PLoS Biol. 2008;6:e159. 18597554 10.1371/journal.pbio.0060159 PMC2443193

[48] Hiser J, Koenigs M. The multifaceted role of the ventromedial prefrontal cortex in emotion, decision making, social cognition, and psychopathology. Biol Psychiatry. 2018;83:638–647. 29275839 10.1016/j.biopsych.2017.10.030 PMC5862740

[49] de la Vega A, Chang LJ, Banich MT, Wager TD, Yarkoni T. Large-scale meta-analysis of human medial frontal cortex reveals tripartite functional organization. J Neurosci. 2016;36:6553–6562. 27307242 10.1523/JNEUROSCI.4402-15.2016 PMC5015787

[50] Doucet G, Naveau M, Petit L, Delcroix N, Zago L, Crivello F, et al. Brain activity at rest: a multiscale hierarchical functional organization. J Neurophysiol. 2011;105:2753–2763. 21430278 10.1152/jn.00895.2010

[51] Glasser MF, Coalson TS, Robinson EC, Hacker CD, Harwell J, Yacoub E, et al. A multi-modal parcellation of human cerebral cortex. Nature. 2016;536:171–178. 27437579 10.1038/nature18933 PMC4990127

[52] Yarkoni T, Poldrack RA, Nichols TE, Van Essen DC, Wager TD. Large-scale automated synthesis of human functional neuroimaging data. Nat Methods. 2011;8:665–670. 21706013 10.1038/nmeth.1635 PMC3146590

[53] Bzdok D, Heeger A, Langner R, Laird AR, Fox PT, Palomero-Gallagher N, et al. Subspecialization in the human posterior medial cortex. Neuroimage. 2015;106:55–71. 25462801 10.1016/j.neuroimage.2014.11.009 PMC4780672

[54] Greicius MD, Supekar K, Menon V, Dougherty RF. Resting-state functional connectivity reflects structural connectivity in the default mode network. Cereb Cortex. 2009;19:72–78. 18403396 10.1093/cercor/bhn059 PMC2605172

[55] Sheline YI, Price JL, Yan Z, Mintun MA. Resting-state functional MRI in depression unmasks increased connectivity between networks via the dorsal nexus. Proc Natl Acad Sci U S A. 2010;107:11020–11025. 20534464 10.1073/pnas.1000446107 PMC2890754

[56] Zhu X, Zhu Q, Shen H, Liao W, Yuan F. Rumination and default mode network subsystems connectivity in first-episode, drug-naive young patients with major depressive disorder. Sci Rep. 2017;7:43105. 28225084 10.1038/srep43105 PMC5320523

[57] Philippi CL, Cornejo MD, Frost CP, Walsh EC, Hoks RM, Birn R, et al. Neural and behavioral correlates of negative self-focused thought associated with depression. Hum Brain Mapp. 2018;39:2246–2257. 29427365 10.1002/hbm.24003 PMC5895491

[58] Bai T, Wei Q, Zu M, Xie W, Wang J, Gong-Jun J, et al. Functional plasticity of the dorsomedial prefrontal cortex in depression reorganized by electroconvulsive therapy: validation in two independent samples. Hum Brain Mapp. 2019;40:465–473. 30240504 10.1002/hbm.24387 PMC6865625

[59] Hagele C, Schlagenhauf F, Rapp M, Sterzer P, Beck A, Bermpohl F, et al. Dimensional psychiatry: reward dysfunction and depressive mood across psychiatric disorders. Psychopharmacology (Berl). 2015;232:331–341. 24973896 10.1007/s00213-014-3662-7 PMC4297301

[60] Mukherjee D, Kable JW. Value-based decision making in mental illness: a meta-analysis. Clin Psychol Sci. 2014;2:767–782.

[61] Christoff K, Irving ZC, Fox KC, Spreng RN, Andrews-Hanna JR. Mind-wandering as spontaneous thought: a dynamic framework. Nat Rev Neurosci. 2016;17:718–731. 27654862 10.1038/nrn.2016.113

[62] Cotter J, Granger K, Backx R, Hobbs M, Looi CY, Barnett JH. Social cognitive dysfunction as a clinical marker: a systematic review of meta-analyses across 30 clinical conditions. Neurosci Biobehav Rev. 2018;84:92–99. 29175518 10.1016/j.neubiorev.2017.11.014

[63] Hoertnagl CM, Hofer A. Social cognition in serious mental illness. Curr Opin Psychiatry. 2014;27:197–202. 24613983 10.1097/YCO.0000000000000055

[64] Derntl B, Habel U. Deficits in social cognition: a marker for psychiatric disorders? Eur Arch Psychiatry Clin Neurosci. 2011;261(suppl 2):S145–S149. 21863344 10.1007/s00406-011-0244-0

[65] Smucny J, Barch DM, Gold JM, Strauss ME, MacDonald AW IIIrd, Boudewyn MA, et al. Cross-diagnostic analysis of cognitive control in mental illness: insights from the CNTRACS consortium. Schizophr Res. 2019;208:377–383. 30704863 10.1016/j.schres.2019.01.018 PMC6544491

[66] Paulus MP. Cognitive control in depression and anxiety: out of control. Curr Opin Behav Sci. 2015;1:113–120.

[67] Dosenbach NU, Nardos B, Cohen AL, Fair DA, Power JD, Church JA, et al. Prediction of individual brain maturity using fMRI. Science. 2010;329:1358–1361. 20829489 10.1126/science.1194144 PMC3135376

[68] Zonneveld HI, Pruim RH, Bos D, Vrooman HA, Muetzel RL, Hofman A, et al. Patterns of functional connectivity in an aging population: the Rotterdam Study. Neuroimage. 2019;189:432–444. 30659958 10.1016/j.neuroimage.2019.01.041

[69] Schilbach L, Hoffstaedter F, Muller V, Cieslik EC, Goya-Maldonado R, Trost S, et al. Transdiagnostic commonalities and differences in resting state functional connectivity of the default mode network in schizophrenia and major depression. Neuroimage Clin. 2016;10:326–335. 26904405 10.1016/j.nicl.2015.11.021 PMC4724692

[70] Gudayol-Ferre E, Pero-Cebollero M, Gonzalez-Garrido AA, Guardia-Olmos J. Changes in brain connectivity related to the treatment of depression measured through fMRI: a systematic review. Front Hum Neurosci. 2015;9:582. 26578927 10.3389/fnhum.2015.00582 PMC4630287

