# Supplementary material for: Transdiagnostic and disease-specific abnormalities in the default-mode network hubs in psychiatric disorders: A meta-analysis of resting-state functional imaging studies
Source: Eur Psychiatry. 2020 May 29;63(1):e57. doi: 10.1192/j.eurpsy.2020.57 (PMC7355168; doi:10.1192/j.eurpsy.2020.57)
Supplement: Supplementary file 1 [file S0924933820000577sup001.docx]

**Transdiagnostic and disease-specific abnormalities in the default-mode network hubs in psychiatric disorders: A meta-analysis of resting-state functional imaging studies**

Gaelle E. Doucet, Delfina Janiri, Rebecca Howard, Madeline O’Brien, Jessica R. Andrews-Hanna, Sophia Frangou

**Supplementary Material**

**Supplementary Methods**

1. **Literature search**

We conducted a systematic literature search in accordance with the Preferred Reporting Items for Systematic Reviews and meta-analyses criteria (http://www.prisma-statement.org/) (Supplementary Figure S1) to identify functional magnetic resonance imaging (fMRI) articles published between January 1, 2005 and January 31, 2019 in PubMed (http://www.pubmed.org), Web of Science (https://apps.webofknowledge.com) and Google Scholar (https://scholar.google.de). The search keywords were “Schizophrenia”, “Major Depressive Disorder”, “Obsessive Compulsive Disorder”, “Unipolar Depression”, “Bipolar Disorder”, “Mania”, “Anxiety Disorder”, “Post Traumatic Stress Disorder”, “Functional”, “Resting-state”, “rest”, “Default-Mode”, “Resting-State Network”, “MRI”, and their various combinations and permutations. Further articles were identified by reference tracing of retrieved papers and previous meta-analyses of functional neuroimaging studies in schizophrenia, major depressive disorder (MDD), bipolar disorder (BD), post-traumatic stress disorder (PTSD), obsessive compulsive disorder (OCD) and anxiety disorders.

1. **Eligibility Criteria for Article Selection**

We included articles that (a) were published between January 1st 2005 and January 31st 2019; (b) examined adults aged 18-65 years; (c) used the diagnostic criteria of the Diagnostic and Statistical Manual of Mental Disorders (DSM) or the International Statistical Classification of Diseases and Related Health Problems; (d) studied healthy individuals and patients with schizophrenia, MDD, BD, OCD, PTSD and anxiety disorders as separate groups; (e) investigated case-control differences in functional connectivity within the whole default-mode network (DMN) or any of its subdivisions^1^, during resting-state conditions; (f) the spatial composition of the DMN was defined as specified by Raichle and colleague^2^ or by Buckner, Andrews-Hanna & Schacter^1^; (g) the temporal correlations between the time-series of distinct DMN brain regions were computed in a time-locked fashion over the entire resting-state scan thus yielding static, non-shifted functional connectivity measures; (h) DMN was extracted using Independent Component Analysis (ICA) or by using a DMN region as a seed^3^; (i) used a whole-brain analyses; (j) reported case-control differences as coordinates in Talairach or Montreal Neurological Institute standard reference space. We excluded articles that (a) did not include a healthy control group; (b) reported on the connectivity of DMN regions during task fMRI; (c) did not perform whole-brain analyses, (e) reported on the connectivity of DMN regions with brain regions outside the DMN. When the same sample was studied longitudinally (either in observational or interventional designs) we only included the baseline findings. When articles reported results from overlapping samples, we included the article with the largest sample size. For PTSD studies we included results originating from the entire sample and not by type of trauma subgroups. Three authors (DJ, GD, RH) independently reviewed all the articles to determine eligibility.

**Supplementary Figure S1. Preferred Reporting Items for Systematic Reviews and meta-analyses (PRISMA) Flow diagram**


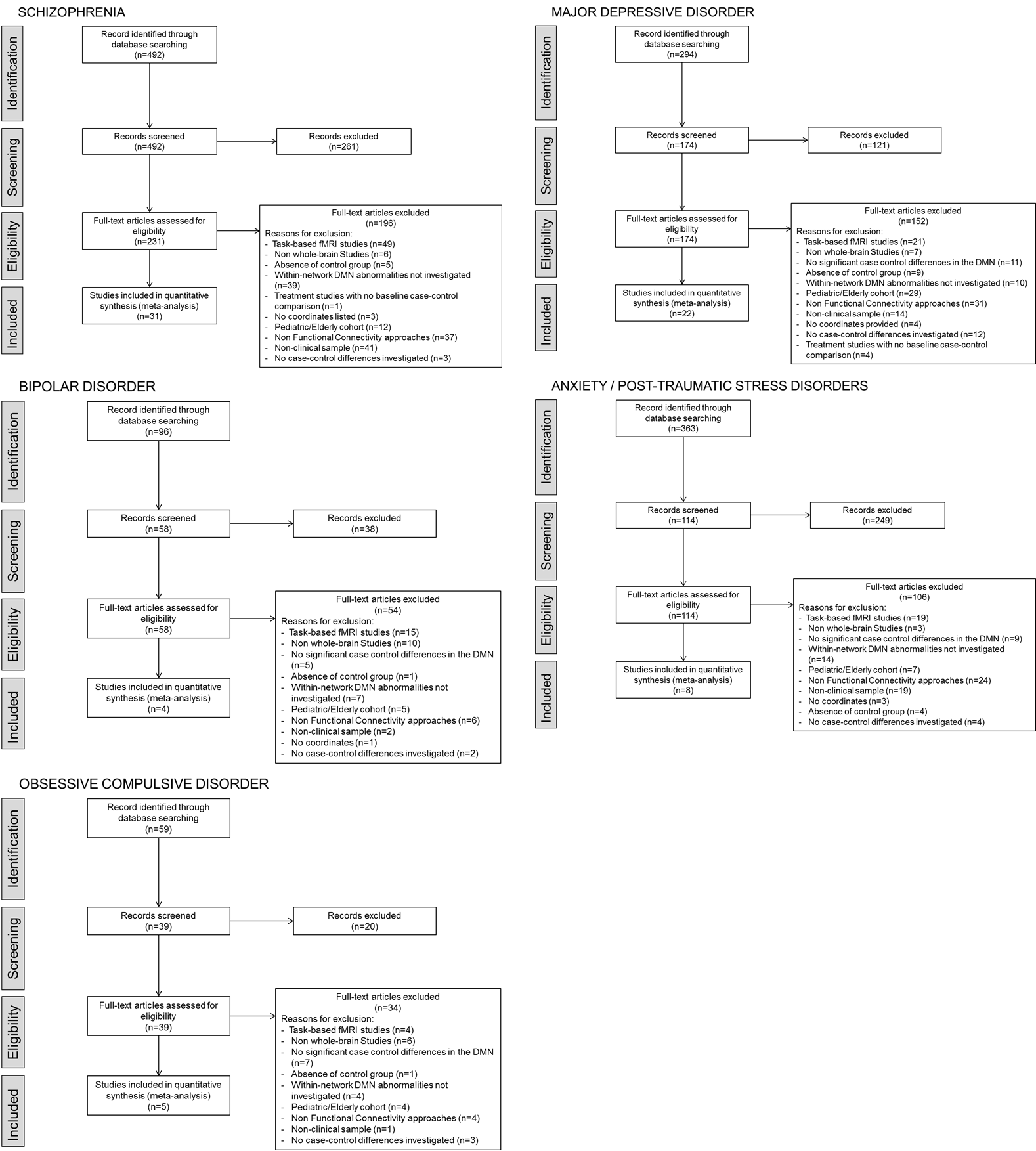


1. **Definition of the Default-Mode Network**

The two most common approaches to reliably identify the DMN have been: (1) independent component analysis (ICA)^4^; and (2) seed-based analyses^3,5^. As per our eligibility criteria, we only considered seed-based analyses in which the seed region was localized within the spatial confines of the DMN as defined by Raichle and colleague^2^ and by Buckner, Andrews-Hanna & Schacter^1^. In the studies selected, seed-regions were most commonly placed at the precuneus (PCu), the posterior cingulate cortex (PCC) or the retrosplenial cortex (55%), followed by seeds in the medial prefrontal cortex (MPFC) and ventral anterior cingulate cortex (vACC) (26%) and while seeds in the hippocampus, and other parts of the temporal cortex were the least used. Of note, the coordinate of the seed was not included in the analyses.

1. **Results of Literature Search**

The 70 articles identified through the literature search comprised 31 articles on schizophrenia, 22 articles on MDD, 4 on BD, 5 on OCD, 7 on PTSD, and 1 on generalized anxiety disorder (Supplementary Table S1). Of the 4 articles on bipolar disorder, 3 included patients with bipolar disorder type I, and 1 included currently depressed patients with bipolar disorder type II.

**5. Full Citation and details of Selected Articles**

| **Supplementary Table S1: Studies Included** | | | | | | | | |
| --- | --- | --- | --- | --- | --- | --- | --- | --- |
| **First Author** | **Year** | **Title** | **Publication** | **PMID** | **Diagnosis** | **Eye condition during acquisition** | **Analytical Approach** | |
| Alonso-Solis | 2016 | Resting-state functional connectivity alterations in the default network of schizophrenia patients with persistent auditory verbal hallucinations | [Schizophr Res. 2015;161(2-3):261-8. doi: 10.1016/j.schres.2014.10.047](https://www.ncbi.nlm.nih.gov/pubmed/25468173) | 25468173 | Schizophrenia | closed | Seed: PCu/PCC  Seed: MPFC  Seed: MTL | |
| Antonucci | 2015 | Association of familial risk for schizophrenia with thalamic and medial prefrontal functional connectivity during attentional control | [Schizophr Res. 2016;173(1-2):23-9. doi: 10.1016/j.schres.2016.03.014](https://www.ncbi.nlm.nih.gov/pubmed/27012899) | 27012899 | Schizophrenia | open | ICA | |
| Bluhm | 2009 | Retrosplenial cortex connectivity in schizophrenia | [Psychiatry Res. 2009;174(1):17-23. doi: 10.1016/j.pscychresns.2009.03.010.](https://www.ncbi.nlm.nih.gov/pubmed/19783410) | 19783410 | Schizophrenia | closed | Seed: Pcu/PCC | |
| Camchong | 2009 | Altered functional and anatomical connectivity in schizophrenia | [Schizophr Bull. 2011;37(3):640-50. doi: 10.1093/schbul/sbp131.](https://www.ncbi.nlm.nih.gov/pubmed/19920062) | 19920062 | Schizophrenia | closed | ICA | |
| Chang | 2014 | Altered default mode and fronto-parietal network subsystems in patients with schizophrenia and their unaffected siblings | [Brain Res. 2014;1562:87-99. doi: 10.1016/j.brainres.2014.03.024.](https://www.ncbi.nlm.nih.gov/pubmed/24675026) | 24675026 | Schizophrenia | closed | ICA | |
| Chen | 2013 | Resting-state fMRI mapping of cerebellar functional dysconnections involving multiple large-scale networks in patients with schizophrenia | [Schizophr Res. 2013;149(1-3):26-34. doi: 10.1016/j.schres.2013.05.029.](https://www.ncbi.nlm.nih.gov/pubmed/23810119) | 23810119 | Schizophrenia | not reported | ICA | |
| Galindo | 2018 | Default Mode Network Aberrant Connectivity Associated with Neurological Soft Signs in Schizophrenia Patients and Unaffected Relatives | [Front Psychiatry. 2018;8:298. doi: 10.3389/fpsyt.2017.00298.](https://www.ncbi.nlm.nih.gov/pubmed/29375404) | 29375404 | Schizophrenia | not reported | Seed: PCu/PCC  Seed: MPFC  ICA | |
| Guo | 2013 | Abnormal default-mode network homogeneity in first-episode, drug-naive schizophrenia at rest | [Prog Neuropsychopharmacol Biol Psychiatry. 2014;49:16-20. doi: 10.1016/j.pnpbp.2013.10.021.](https://www.ncbi.nlm.nih.gov/pubmed/24216538) | 24216538 | Schizophrenia | closed | ICA | |
| Guo | 2015 | Resting-state cerebellar-cerebral networks are differently affected in first-episode, drug-naive schizophrenia patients and unaffected siblings | [Sci Rep. 2015;5:17275. doi: 10.1038/srep17275](https://www.ncbi.nlm.nih.gov/pubmed/26608842) | 26608842 | Schizophrenia | closed | Seed: PCu/PCC | |
| Guo | 2017 | Olanzapine modulates the default-mode network homogeneity in recurrent drug-free schizophrenia at rest | [Aust N Z J Psychiatry. 2017;51(10):1000-1009. doi: 10.1177/0004867417714952.](https://www.ncbi.nlm.nih.gov/pubmed/28605934) | 28605934 | Schizophrenia | not reported | ICA | |
| Guo | 2017 | Hyperactivity of the default-mode network in first-episode, drug-naive schizophrenia at rest revealed by family-based case-control and traditional case-control designs | [Medicine (Baltimore). 2017;96(13):e6223. doi: 10.1097/MD.0000000000006223](https://www.ncbi.nlm.nih.gov/pubmed/28353559) | 28353559 | Schizophrenia | not reported | ICA | |
| He | 2013 | Aberrant intrinsic brain activity and cognitive deficit in first-episode treatment-naive patients with schizophrenia | [Psychol Med. 2013;43(4):769-80. doi: 10.1017/S0033291712001638.](https://www.ncbi.nlm.nih.gov/pubmed/22883428) | 22883428 | Schizophrenia | closed | Seed: PCu/PCC | |
| Jiang | 2017 | Common and distinct dysfunctional patterns contribute to triple network model in schizophrenia and depression: A preliminary study | [Prog Neuropsychopharmacol Biol Psychiatry. 2017;79(Pt B):302-310. doi: 10.1016/j.pnpbp.2017.07.007.](https://www.ncbi.nlm.nih.gov/pubmed/28705767) | 28705767 | Schizophrenia | closed | Seed: PCu/PCC | |
| Khadka | 2013 | Is aberrant functional connectivity a psychosis endophenotype? A resting state functional magnetic resonance imaging study | [Biol Psychiatry. 2013;74(6):458-66. doi: 10.1016/j.biopsych.2013.04.024.](https://www.ncbi.nlm.nih.gov/pubmed/23746539) | 23746539 | Schizophrenia | open | ICA | |
| Kraguljac | 2014 | Abnormalities in large scale functional networks in unmedicated patients with schizophrenia and effects of risperidone | [Neuroimage Clin. 2015;10:146-58. doi: 10.1016/j.nicl.2015.11.015.](https://www.ncbi.nlm.nih.gov/pubmed/26793436) | 26793436 | Schizophrenia | open | Seed: MTL | |
| Li | 2015 | A splitting brain: Imbalanced neural networks in schizophrenia | [Psychiatry Res. 2015;232(2):145-53. doi: 10.1016/j.pscychresns.2015.03.001.](https://www.ncbi.nlm.nih.gov/pubmed/25819347) | 25819347 | Schizophrenia | closed | Seed: PCu/PCC  Seed: MPFC | |
| Mallikarjun | 2018 | Aberrant salience network functional connectivity in auditory verbal hallucinations: a first episode psychosis sample | [Transl Psychiatry. 2018;8(1):69. doi: 10.1038/s41398-018-0118-6](https://www.ncbi.nlm.nih.gov/pubmed/29581420) | 29581420 | Schizophrenia | closed | Seed: lateral temporal cortex | |
| Manoliu | 2014 | Aberrant dependence of default mode/central executive network interactions on anterior insular salience network activity in schizophrenia | [Schizophr Bull. 2014;40(2):428-37. doi: 10.1093/schbul/sbt037.](https://www.ncbi.nlm.nih.gov/pubmed/23519021) | 23519021 | Schizophrenia | closed | ICA | |
| Meda | 2014 | Multivariate analysis reveals genetic associations of the resting default mode network in psychotic bipolar disorder and schizophrenia | [Proc Natl Acad Sci U S A. 2014;111(19):E2066-75. doi: 10.1073/pnas.1313093111.](https://www.ncbi.nlm.nih.gov/pubmed/24778245) | 24778245 | Schizophrenia | open | ICA | |
| Mingoia | 2012 | Default mode network activity in schizophrenia studied at resting state using probabilistic ICA | [Schizophr Res. 2012;138(2-3):143-9. doi: 10.1016/j.schres.2012.01.036.](https://www.ncbi.nlm.nih.gov/pubmed/22578721) | 22578721 | Schizophrenia | closed | ICA | |
| Moran | 2013 | Disruption of anterior insula modulation of large-scale brain networks in schizophrenia | [Biol Psychiatry. 2013;74(6):467-74. doi: 10.1016/j.biopsych.2013.02.029.](https://www.ncbi.nlm.nih.gov/pubmed/23623456) | 23623456 | Schizophrenia | open | Seed: PCu/PCC | |
| Ongur | 2010 | Default mode network abnormalities in bipolar disorder and schizophrenia | [Psychiatry Res. 2010;183(1):59-68. doi: 10.1016/j.pscychresns.2010.04.008.](https://www.ncbi.nlm.nih.gov/pubmed/20553873) | 20553873 | Schizophrenia | open | ICA | |
| Orliac | 2013 | Links among resting-state default-mode network, salience network, and symptomatology in schizophrenia | [Schizophr Res. 2013;148(1-3):74-80. doi: 10.1016/j.schres.2013.05.007.](https://www.ncbi.nlm.nih.gov/pubmed/23727217) | 23727217 | Schizophrenia | closed | ICA | |
| Rotarska-Jagiela | 2010 | Resting-state functional network correlates of psychotic symptoms in schizophrenia | [Schizophr Res. 2010;117(1):21-30. doi: 10.1016/j.schres.2010.01.001.](https://www.ncbi.nlm.nih.gov/pubmed/20097544) | 20097544 | Schizophrenia | open | ICA | |
| Schilbach | 2016 | Transdiagnostic commonalities and differences in resting state functional connectivity of the default mode network in schizophrenia and major depression | [Neuroimage Clin. 2015;10:326-35. doi: 10.1016/j.nicl.2015.11.021.](https://www.ncbi.nlm.nih.gov/pubmed/26904405) | 26904405 | Schizophrenia | closed | Seed: PCu/PCC  Seed: MPFC | |
| Sharma | 2018 | Altered resting state functional connectivity in early course schizophrenia | [Psychiatry Res Neuroimaging. 2018;271:17-23. doi: 10.1016/j.pscychresns.2017.11.013.](https://www.ncbi.nlm.nih.gov/pubmed/29220695) | 29220695 | Schizophrenia | closed | ICA | |
| Whitfield-Gabrieli | 2009 | Hyperactivity and hyperconnectivity of the default network in schizophrenia and in first-degree relatives of persons with schizophrenia | [Proc Natl Acad Sci U S A. 2009;106(4):1279-84. doi: 10.1073/pnas.0809141106.](https://www.ncbi.nlm.nih.gov/pubmed/19164577) | 19164577 | Schizophrenia | not reported | Seed: PCu/PCC  Seed: MPFC | |
| Woodward | 2011 | Functional resting-state networks are differentially affected in schizophrenia | [Schizophr Res. 2011;130(1-3):86-93. doi: 10.1016/j.schres.2011.03.010.](https://www.ncbi.nlm.nih.gov/pubmed/21458238) | 21458238 | Schizophrenia | closed | Seed: PCu/PCC | |
| Zhou | 2016 | Inefficient DMN Suppression in Schizophrenia Patients with Impaired Cognitive Function but not Patients with Preserved Cognitive Function | [Sci Rep. 2016;6:21657. doi: 10.1038/srep21657](https://www.ncbi.nlm.nih.gov/pubmed/26882844) | 26882844 | Schizophrenia | closed | ICA | |
| Zhou | 2019 | Convergent and divergent altered patterns of default mode network in deficit and non-deficit schizophrenia | [Prog Neuropsychopharmacol Biol Psychiatry. 2019;89:427-434. doi: 10.1016/j.pnpbp.2018.10.012.](https://www.ncbi.nlm.nih.gov/pubmed/30367960) | 30367960 | Schizophrenia | closed | ICA | |
| Zong | 2018 | A Dissociation in Effects of Risperidone Monotherapy on Functional and Anatomical Connectivity Within the Default Mode Network | [Schizophr Bull. 2018; doi: 10.1093/schbul/sby175.](https://www.ncbi.nlm.nih.gov/pubmed/30508134) | 30508134 | Schizophrenia | closed | ICA | |
| Bessette | 2018 | Reliability, Convergent Validity and Time Invariance of Default Mode Network Deviations in Early Adult Major Depressive Disorder | [Front Psychiatry. 2018;9:244. doi: 10.3389/fpsyt.2018.00244. eCollection 2018](https://www.ncbi.nlm.nih.gov/pubmed/29937738) | 29937738 | MDD | open | Seed: PCu/PCC  Seed: MTL |  |
| Bluhm | 2009 | Resting state default-mode network connectivity in early depression using a seed region-of-interest analysis: decreased connectivity with caudate nucleus | [Psychiatry Clin Neurosci. 2009;63(6):754-61. doi: 10.1111/j.1440-1819.2009.02030.x.](https://www.ncbi.nlm.nih.gov/pubmed/20021629) | 20021629 | MDD | closed | Seed: PCu/PCC |  |
| Chen | 2015 | Aberrant connectivity within the default mode network in first-episode, treatment-naïve major depressive disorder | [J Affect Disord. 2015;183:49-56. doi: 10.1016/j.jad.2015.04.052.](https://www.ncbi.nlm.nih.gov/pubmed/26001663) | 26001663 | MDD | closed | Seed: PCu/PCC |  |
| Crowther | 2015 | Resting-state connectivity predictors of response to psychotherapy in major depressive disorder | [Neuropsychopharmacology. 2015 Jun;40(7):1659-73. doi: 10.1038/npp.2015.12.](https://www.ncbi.nlm.nih.gov/pubmed/25578796) | 25578796 | MDD | open | Seed: PCu/PCC |  |
| de Kwaasteniet | 2015 | Decreased Resting-State Connectivity between Neurocognitive Networks in Treatment Resistant Depression | [Front Psychiatry. 2015;6:28. doi: 10.3389/fpsyt.2015.00028.](https://www.ncbi.nlm.nih.gov/pubmed/25784881) | 25784881 | MDD | open | Seed: MPFC |  |
| Deng | 2016 | Modulation of the Default Mode Network in First-Episode, Drug-Naïve Major Depressive Disorder via Acupuncture at Baihui (GV20) Acupoint | [Front Hum Neurosci. 2016;10:230. doi: 10.3389/fnhum.2016.00230.](https://www.ncbi.nlm.nih.gov/pubmed/27242492) | 27242492 | MDD | closed | Seed: PCu/PCC |  |
| Evans | 2018 | Default Mode Connectivity in Major Depressive Disorder Measured Up to 10 Days After Ketamine Administration | [Biol Psychiatry. 2018;84(8):582-590. doi: 10.1016/j.biopsych.2018.01.027.](https://www.ncbi.nlm.nih.gov/pubmed/29580569) | 29580569 | MDD | closed | Seed: PCu/PCC |  |
| Gao | 2018 | Abnormal Default Mode Network Homogeneity in Treatment-Naive Patients With First-Episode Depression | [Front Psychiatry. 2018;9:697. doi: 10.3389/fpsyt.2018.00697.](https://www.ncbi.nlm.nih.gov/pubmed/30618871) | 30618871 | MDD | closed | ICA |  |
| Goya-Maldonado | 2016 | Differentiating unipolar and bipolar depression by alterations in large-scale brain networks | [Hum Brain Mapp. 2016;37(2):808-18. doi: 10.1002/hbm.23070.](https://www.ncbi.nlm.nih.gov/pubmed/26611711) | 26611711 | MDD | open | ICA |  |
| Greicius | 2007 | Resting-state functional connectivity in major depression: abnormally increased contributions from subgenual cingulate cortex and thalamus | [Biol Psychiatry. 2007;62(5):429-37.](https://www.ncbi.nlm.nih.gov/pubmed/17210143) | 17210143 | MDD | closed | ICA |  |
| Guo | 2014 | Abnormal default-mode network homogeneity in first-episode, drug-naive major depressive disorder | [PLoS One. 2014;9(3):e91102. doi: 10.1371/journal.pone.0091102.](https://www.ncbi.nlm.nih.gov/pubmed/24609111) | 24609111 | MDD | closed | ICA |  |
| Guo | 2018 | Increased anterior default-mode network homogeneity in first-episode, drug-naive major depressive disorder: A replication study | [J Affect Disord. 2018;225:767-772. doi: 10.1016/j.jad.2017.08.089.](https://www.ncbi.nlm.nih.gov/pubmed/28938513) | 28938513 | MDD | closed | ICA |  |
| Guo | 2018 | Increased anterior default-mode network homogeneity in first-episode, drug-naive major depressive disorder: A replication study | [J Affect Disord. 2018;225:767-772. doi: 10.1016/j.jad.2017.08.089.](https://www.ncbi.nlm.nih.gov/pubmed/28938513) | 28938513 | MDD | closed | ICA |  |
| Jacobs | 2016 | Decoupling of the amygdala to other salience network regions in adolescent-onset recurrent major depressive disorder | [Psychol Med. 2016;46(5):1055-67. doi: 10.1017/S0033291715002615. Epub 2016 Jan 20](https://www.ncbi.nlm.nih.gov/pubmed/26784396) | 26784396 | MDD | open | Seed: PCu/PCC |  |
| Li | 2017 | Abnormal resting state effective connectivity within the default mode network in major depressive disorder: A spectral dynamic causal modeling study | [Brain Behav. 2017;7(7):e00732. doi: 10.1002/brb3.732.](https://www.ncbi.nlm.nih.gov/pubmed/28729938) | 28729938 | MDD | closed | ICA |  |
| Manoliu | 2014 | Insular dysfunction within the salience network is associated with severity of symptoms and aberrant inter-network connectivity in major depressive disorder | [Front Hum Neurosci. 2014;7:930. doi: 10.3389/fnhum.2013.00930.](https://www.ncbi.nlm.nih.gov/pubmed/24478665) | 24478665 | MDD | closed | ICA |  |
| Parlar | 2016 | Relation between patterns of intrinsic network connectivity, cognitive functioning, and symptom presentation in trauma-exposed patients with major depressive disorder | [Brain Behav. 2017;7(5):e00664. doi: 10.1002/brb3.664.](https://www.ncbi.nlm.nih.gov/pubmed/28523217) | 28523217 | MDD | open | ICA |  |
| Renner | 2017 | Negative mood-induction modulates default mode network resting-state functional connectivity in chronic depression | [J Affect Disord. 2017;208:590-596. doi: 10.1016/j.jad.2016.10.022.](https://www.ncbi.nlm.nih.gov/pubmed/27810271) | 27810271 | MDD | not recorded | Seed: PCu/PCC |  |
| Sambataro | 2014 | Revisiting default mode network function in major depression: evidence for disrupted subsystem connectivity | [Psychol Med. 2014;44(10):2041-51. doi: 10.1017/S0033291713002596.](https://www.ncbi.nlm.nih.gov/pubmed/24176176) | 24176176 | MDD | closed | ICA |  |
| Sambataro | 2017 | Altered dynamics of brain connectivity in major depressive disorder at-rest and during task performance | [Psychiatry Res Neuroimaging. 2017;259:1-9. doi: 10.1016/j.pscychresns.2016.11.001.](https://www.ncbi.nlm.nih.gov/pubmed/27918910) | 27918910 | MDD | closed | ICA |  |
| Sawaya | 2015 | Resting-state functional connectivity of antero-medial prefrontal cortex sub-regions in major depression and relationship to emotional intelligence | [Int J Neuropsychopharmacol. 2015;18(6). pii: pyu112. doi: 10.1093/ijnp/pyu112](https://www.ncbi.nlm.nih.gov/pubmed/25744282) | 25744282 | MDD | open | Seed: MPFC |  |
| Schilbach | 2016 | Transdiagnostic commonalities and differences in resting state functional connectivity of the default mode network in schizophrenia and major depression | [Neuroimage Clin. 2015;10:326-35. doi: 10.1016/j.nicl.2015.11.021.](https://www.ncbi.nlm.nih.gov/pubmed/26904405) | 26904405 | MDD | closed | Seed: PCu/PCC  Seed: MPFC |  |
| Zhu | 2012 | Evidence of a dissociation pattern in resting-state default mode network connectivity in first-episode, treatment-naive major depression patients | [Biol Psychiatry. 2012;71(7):611-7. doi: 10.1016/j.biopsych.2011.10.035.](https://www.ncbi.nlm.nih.gov/pubmed/22177602) | 22177602 | MDD | closed | ICA |  |
| Gong | 2019 | Disrupted functional connectivity within the default mode network and salience network in unmedicated bipolar II disorder | [Prog Neuropsychopharmacol Biol Psychiatry. 2019;88:11-18. doi: 10.1016/j.pnpbp.2018.06.012.](https://www.ncbi.nlm.nih.gov/pubmed/29958116) | 29958116 | BD | closed | Seed: PCu/PCC |  |
| Magioncalda | 2015 | Functional connectivity and neuronal variability of resting state activity in bipolar disorder--reduction and decoupling in anterior cortical midline structures | [Hum Brain Mapp. 2015;36(2):666-82. doi: 10.1002/hbm.22655.](https://www.ncbi.nlm.nih.gov/pubmed/25307723) | 25307723 | BD | closed | Seed: PCu/PCC |  |
| Ongur | 2010 | Default mode network abnormalities in bipolar disorder and schizophrenia | [Psychiatry Res. 2010;183(1):59-68. doi: 10.1016/j.pscychresns.2010.04.008.](https://www.ncbi.nlm.nih.gov/pubmed/20553873) | 20553873 | BD | open | ICA |  |
| Wang | 2018 | Shared and specific functional connectivity alterations in unmedicated bipolar and major depressive disorders based on the triple-network model | [Brain Imaging Behav. 2018;. doi: 10.1007/s11682-018-9978-x.](https://www.ncbi.nlm.nih.gov/pubmed/30382529) | 30382529 | BD | closed | ICA |  |
| DiGangi | 2016 | Reduced default mode network connectivity following combat trauma | [Neurosci Lett. 2016;615:37-43. doi: 10.1016/j.neulet.2016.01.010.](https://www.ncbi.nlm.nih.gov/pubmed/26797653) | 26797653 | PTSD | open | Seed: PCu/PCC  Seed: MPFC |  |
| Ke | 2018 | Typhoon-Related Post-Traumatic Stress Disorder and Trauma Might Lead to Functional Integration Abnormalities in Intra- and Inter-Resting State Networks: a Resting-State Fmri Independent Component Analysis | [Cell Physiol Biochem. 2018;48(1):99-110. doi: 10.1159/000491666.](https://www.ncbi.nlm.nih.gov/pubmed/30001548) | 30001548 | PTSD | closed | ICA |  |
| Liao | 2010 | Selective aberrant functional connectivity of resting state networks in social anxiety disorder | [Neuroimage. 2010;52(4):1549-58. doi: 10.1016/j.neuroimage.2010.05.010.](https://www.ncbi.nlm.nih.gov/pubmed/20470894) | 20470894 | Social Anxiety Disorder | closed | ICA |  |
| Miller | 2017 | Default Mode Network Subsystems are Differentially Disrupted in Posttraumatic Stress Disorder | [Biol Psychiatry Cogn Neurosci Neuroimaging. 2017;2(4):363-371.](https://www.ncbi.nlm.nih.gov/pubmed/28435932) | 28435932 | PTSD | open | Seed: PCu/PCC  Seed: MPFC |  |
| Olson | 2018 | Regional Prefrontal Resting-State Functional Connectivity in Posttraumatic Stress Disorder | [Biol Psychiatry Cogn Neurosci Neuroimaging. 2019;4(4):390-398. doi: 10.1016/j.bpsc.2018.09.012.](https://www.ncbi.nlm.nih.gov/pubmed/30449518) | 30449518 | PTSD | open | Seed: MPFC |  |
| Shang | 2014 | Alterations in low-level perceptual networks related to clinical severity in PTSD after an earthquake: a resting-state fMRI study | [PLoS One. 2014;9(5):e96834. doi: 10.1371/journal.pone.0096834.](https://www.ncbi.nlm.nih.gov/pubmed/24823717) | 24823717 | PTSD | closed | ICA |  |
| Sripada | 2012 | Neural dysregulation in posttraumatic stress disorder: evidence for disrupted equilibrium between salience and default mode brain networks | [Psychosom Med. 2012;74(9):904-11. doi: 10.1097/PSY.0b013e318273bf33.](https://www.ncbi.nlm.nih.gov/pubmed/23115342) | 23115342 | PTSD | open | Seed: PCu/PCC  Seed: MPFC |  |
| Zhang | 2015 | Intranetwork and internetwork functional connectivity alterations in post-traumatic stress disorder | [J Affect Disord. 2015;187:114-21. doi: 10.1016/j.jad.2015.08.043.](https://www.ncbi.nlm.nih.gov/pubmed/26331685) | 26331685 | PTSD | closed | ICA |  |
| Beucke | 2014 | Default mode network subsystem alterations in obsessive-compulsive disorder | [Br J Psychiatry. 2014;205(5):376-82. doi: 10.1192/bjp.bp.113.137380.](https://www.ncbi.nlm.nih.gov/pubmed/25257066) | 25257066 | OCD | closed | Seed: PCu/PCC  Seed: MPFC  Seed: lateral temporal cortex  Seed: IPL  Seed: MTL |  |
| Chen | 2019 | Altered network homogeneity of the default-mode network in drug-naive obsessive-compulsive disorder | Prog Neuropsychopharmacol Biol Psychiatry. 2019;93:77-83. doi: 10.1016/j.pnpbp.2019.03.008. [ | 30905622 | OCD | closed | ICA |  |
| Fan | 2017 | Altered connectivity within and between the default mode, central executive, and salience networks in obsessive-compulsive disorder | [J Affect Disord. 2017;223:106-114. doi: 10.1016/j.jad.2017.07.041.](https://www.ncbi.nlm.nih.gov/pubmed/28743059) | 28743059 | OCD | closed | ICA |  |
| Hou | 2013 | Morphologic and Functional Connectivity Alterations of Corticostriatal and Default Mode Network in Treatment-Naïve Patients with Obsessive-Compulsive Disorder | [PLoS One.](https://www.ncbi.nlm.nih.gov/pubmed/24358320) 2013;8(12):e83931. doi: 10.1371/journal.pone.0083931 | 24358320 | OCD | closed | Seed: PCu/PCC  Seed: MPFC |  |
| Jang | 2010 | Functional connectivity in fronto-subcortical circuitry during the resting state in obsessive-compulsive disorder | Neurosci Lett. 2010;474(3):158-162. doi: 10.1016/j.neulet.2010.03.031. | 20302914 | OCD | closed | Seed: PCu/PCC |  |
| Abbreviations: BD=Bipolar Disorder; ICA: Independent component analysis;IPL=inferior parietal lobule; MDD=Major Depressive Disorder; MTL= hippocampus/parahippocampus; MPFC=medial prefrontal cortex including the Anterior Cingulate Cortex; PCu= precuneus, OCD=Obsessive Compulsive Disorder; PCC=posterior cingulate cortex including the retrosplenial cortex; PTSD=Posttraumatic Stress Disorder. | | | | | | | |  |

**6. Data extraction and coding**

Coordinates extracted from the original articles were coded with respect to diagnosis, the direction of change in connectivity in patients relative to the healthy control group (i.e., hypo- or hyper-connectivity), the strength of the magnetic field of the scanner, the analytical method used to compute inter-regional correlations (ICA or seed-based), and according to whether participants were instructed to keep their eyes open or closed during data acquisition. From each article and separately for patients and control, we recorded the number of participants, mean age and percentage (%) male. For patients only, the medication status (% receiving any psychotropic medication) and symptom severity were also recorded. Patient’s symptom severity was based on the mean score of the instrument used to rate symptoms in the primary study. When multiple instruments were used, we extracted only the mean value and standard-deviation (SD) of the instrument that was most commonly employed in all other studies. To accommodate the various instruments across disorders, their rating was scored as “minimal”, “mild”, “moderate” and “severe”. For most scales, the score was recoded based on instrument’s manual. For all other scales we used the quartile scores.

**7. Activation Likelihood Estimation (ALE)**

The ALE algorithm tested whether the spatial distribution within the brain of the peaks (foci) from the coordinates sets included in the meta-analysis differed from a random distribution ^6-8^. When needed, coordinates were transformed from Montreal Neurology Institute space to Talairach space using the icbm_other2tal transformation. Foci of each coordinates set were modelled as centers of a 3D Gaussian distribution accounting for the uncertainty associated with each focus. The full-width-at-half-maximum of these probability distributions was based on empirical data from the between-subject and between-template variance. The between-subject variance was weighted by the size of the sample, so that findings from larger samples had higher localizing power. The probabilities of all foci associated with each coordinates sets are aggregated using the highest probability of each voxel at any focus reported for that sets. This approach ensures that foci that are in close vicinity do not exert a cumulative influence on probability values. A modeled voxel-wise activation map is then created for each coordinates set. The combination of all activation maps yields voxel-wise ALE scores that describe the convergence of results at each location of the brain. To distinguish true spatial convergence across coordinates sets from random overlap, a random effects model was used to compare ALE scores against an analytically derived null-distribution map. The p-value of a given voxel-wise ALE score represents the proportion of equal or higher values obtained under the null-distribution. The resulting non-parametric uncorrected voxel-wise p-values were thresholded at the cluster-forming threshold of *P*<0.001. Then the size of the clusters surviving this threshold is compared against a null-distribution of cluster-sizes derived by simulating 5000 datasets of randomly distributed foci but with otherwise identical properties (number of foci, uncertainty) as the original dataset. Family-wise error correction at P<0.05 was then applied to this distribution to identify clusters of coordinates that only exceed in 5 % of all random simulations

**Supplementary Results**

**1. Analysis of unmedicated patients**


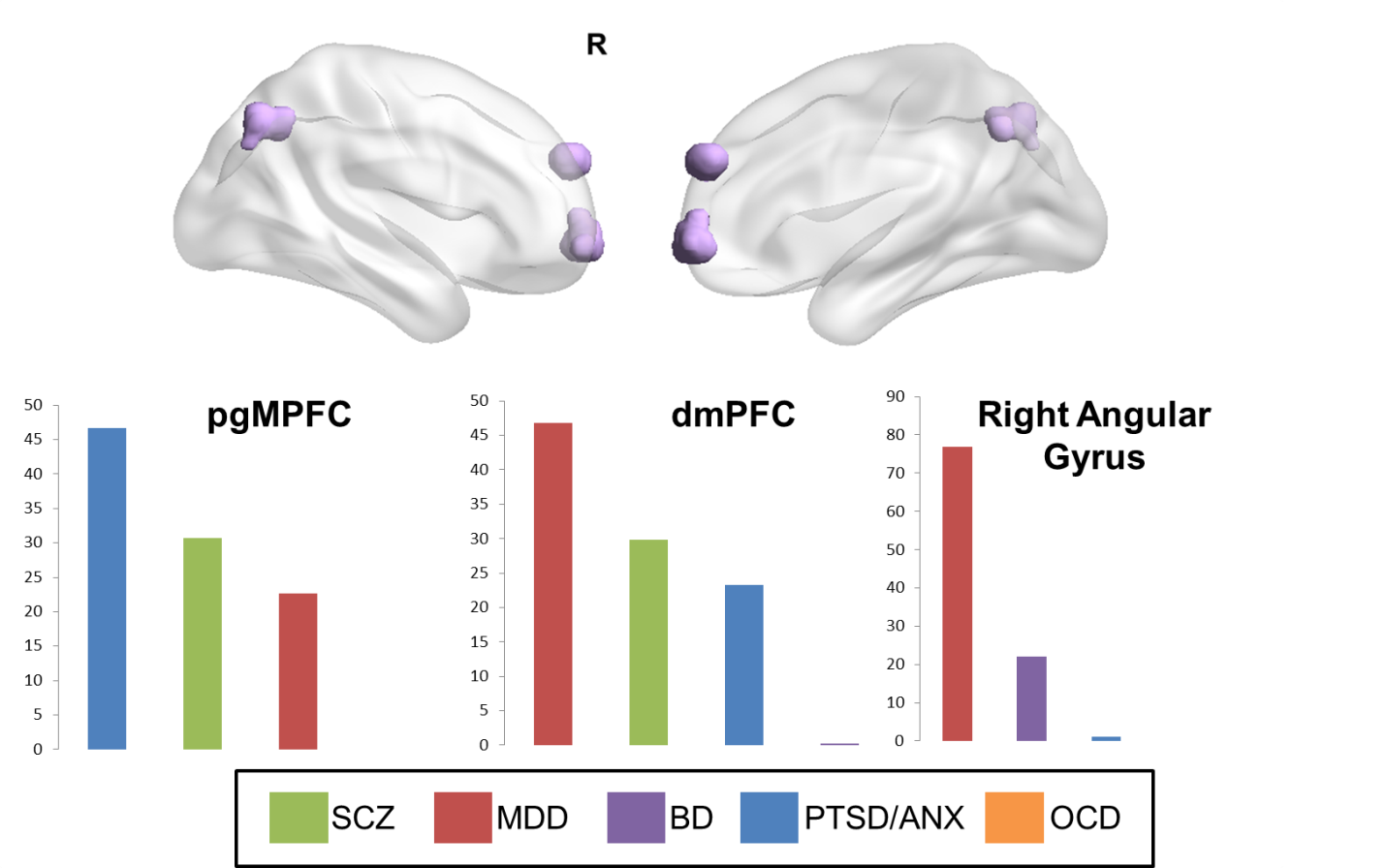


**Supplementary Figure S2: Transdiagnostic Clusters of aberrant connectivity in the Default Mode Network in unmedicated patients.** SCZ: Schizophrenia, MDD: Major Depressive Disorder, BD: Bipolar Disorder, PTSD: Posttraumatic Stress Disorder, ANX: Social Anxiety, OCD: Obsessive Compulsive Disorder, dmPFC: dorsomedial prefrontal cortex, pgMPFC: perigenual medial prefrontal cortex.

**Supplementary References**

1 Buckner, R. L., Andrews-Hanna, J. R. & Schacter, D. L. The brain's default network: anatomy, function, and relevance to disease. *Annals of the New York Academy of Sciences* **1124**, 1-38, doi:10.1196/annals.1440.011 (2008).

2 Raichle, M. E. *et al.* A default mode of brain function. *Proceedings of the National Academy of Sciences of the United States of America* **98**, 676-682, doi:10.1073/pnas.98.2.676 (2001).

3 Fox, M. D. *et al.* The human brain is intrinsically organized into dynamic, anticorrelated functional networks. *Proceedings of the National Academy of Sciences of the United States of America* **102**, 9673-9678, doi:10.1073/pnas.0504136102 (2005).

4 McKeown, M. J. & Sejnowski, T. J. Independent component analysis of fMRI data: examining the assumptions. *Human brain mapping* **6**, 368-372 (1998).

5 Biswal, B., Yetkin, F. Z., Haughton, V. M. & Hyde, J. S. Functional connectivity in the motor cortex of resting human brain using echo-planar MRI. *Magn Reson Med* **34**, 537-541 (1995).

6 Eickhoff, S. B., Bzdok, D., Laird, A. R., Kurth, F. & Fox, P. T. Activation likelihood estimation meta-analysis revisited. *NeuroImage* **59**, 2349-2361, doi:10.1016/j.neuroimage.2011.09.017 (2012).

7 Eickhoff, S. B. *et al.* Coordinate-based activation likelihood estimation meta-analysis of neuroimaging data: a random-effects approach based on empirical estimates of spatial uncertainty. *Human brain mapping* **30**, 2907-2926, doi:10.1002/hbm.20718 (2009).

8 Eickhoff, S. B. *et al.* Behavior, sensitivity, and power of activation likelihood estimation characterized by massive empirical simulation. *NeuroImage* **137**, 70-85, doi:10.1016/j.neuroimage.2016.04.072 (2016).
